# Association Studies and Genomic Prediction for Genetic Improvements in Agriculture

**DOI:** 10.3389/fpls.2022.904230

**Published:** 2022-06-02

**Authors:** Qianqian Zhang, Qin Zhang, Just Jensen

**Affiliations:** ^1^Institute of Biotechnology, Beijing Academy of Agricultural and Forestry Sciences, Beijing, China; ^2^College of Animal Science and Technology, Shandong Agricultural University, Taian, China; ^3^College of Animal Science and Technology, China Agricultural University, Beijing China; ^4^Centre for Quantitative Genetics and Genomics, Aarhus University, Aarhus, Denmark

**Keywords:** agriculture, genome-wide association study, genomic prediction, breeding, genetic improvement

## Abstract

To feed the fast growing global population with sufficient food using limited global resources, it is urgent to develop and utilize cutting-edge technologies and improve efficiency of agricultural production. In this review, we specifically introduce the concepts, theories, methods, applications and future implications of association studies and predicting unknown genetic value or future phenotypic events using genomics in the area of breeding in agriculture. Genome wide association studies can identify the quantitative genetic loci associated with phenotypes of importance in agriculture, while genomic prediction utilizes individual genetic value to rank selection candidates to improve the next generation of plants or animals. These technologies and methods have improved the efficiency of genetic improvement programs for agricultural production *via* elite animal breeds and plant varieties. With the development of new data acquisition technologies, there will be more and more data collected from high-through-put technologies to assist agricultural breeding. It will be crucial to extract useful information among these large amounts of data and to face this challenge, more efficient algorithms need to be developed and utilized for analyzing these data. Such development will require knowledge from multiple disciplines of research.

## Introduction

Genome wide association study (GWAS) is used to find associations between specific genotypes obtained from direct measurements on DNA level and phenotype using a specific statistical or mathematical method which can identify the correlation or connection between genotype and phenotype. It is methods of great importance in the areas of animal and plant breeding. Identifying areas on the genome with effects on the phenotype such as yield or physiological (Jannink et al., 2010; Crossa et al., 2017) can be used to identify individuals or lines with better yield or better adaptation to current and future climate conditions than previous individuals or lines. These information could be used to understand the regulations or predict the regulations between genes toward phenotypes. Therefore, the identified genetic markers correlated with or underlying the genes affecting the phenotypes from genome-wide association studies can be further utilized in plant or animal breeding. Collectively, these markers can be used to predict the expected phenotype (expected breeding values, i.e., EBV) of varieties or lines for precision plant or animal breeding (Meuwissen et al., 2001; Meuwissen and Goddard, 2010; Daetwyler et al., 2014; van Binsbergen et al., 2015). The EBV of a variety or line is the expected values of the genes carried when these genes are transmitted to offspring. The estimation of EBV is basis of genomic selection and the improved accuracy of EBV estimation can be directly translated into big difference in genetic gain.

GWAS has commonly been used in identifying genes or genotypes affecting specific phenotypes (traits) in agriculture. Usually, researchers use general linear regression to identify the relation between genotypes and phenotypes and the general solutions can be found using least squares. In plant or animal breeding, the genes or genotypes associated with phenotypes can be utilized to study the function or classifying plants or animals into simple classes when the phenotypic effect of genes or genotypes is relatively large and the number of genes significantly associated with the phenotypes is relatively small (Daetwyler et al., 2014; Zhang et al., 2016b). The genotype or genes with large phenotypic effect are often studied in depth in order to breed better breeds or varieties with better production and disease resistance toward the breeding objectives in plants and animals through functional studies (Brondum et al., 2015; Zhang et al., 2018). These information derived from GWAS analysis can also provide prior information or the information used for variants pruning in genomic prediction.

Notably, association between genotypes and phenotypes does not always reflect the causal relationship between genotype and phenotype because the correlated structures of genotypes and phenotypes are very complex and detected loci are mostly in linkage disequilibrium with the causal loci. In order to explore and utilize the complex structure of the genotype and phenotypes better, more complex model is used for GWAS, for example, random regression model, mixed linear models etc. So far, very many genes with small effects have been identified in many agricultural species and there are extensive databases classifying these effects into different categories (Gibson, 2012; de los Campos et al., 2015). During the long-term selection since domestication of many agricultural species, many of these genes with relatively large effects have been fixed by various forms of selection. For example, rice species have been domesticated in China since 10,000 years ago and the following conscious or unconscious selection have fixed most genes of large effects. However, even though, there are still very considerable amounts of genetic variation in all agricultural species which is primarily due to very many genes each with small effects that collectively contribute to the phenotypic variation. These small effects are very difficult to be detected and validated experimentally because very large experimental populations are needed. Phenotypic testing and extensive dense genotyping instead should be used to predict the collective effects of these genes with small effects still segregating in the corresponding agricultural species instead of testing for each single gene. Meanwhile, when performing GWAS study, the same genotypes have different effect sizes when associating across various phenotypes. This reflects that the genetic architectures underlying different phenotypes are complex, correlated and interactive defined as pleiotropy of the genetic architectures or background of different phenotypes (Daetwyler et al., 2014). In plants, the same genotypes have different effects sizes even for the same phenotype and this has resulted from the significant genotype by environment interaction when the plants are grown in different environments (Campbell and Waser, 2001). Therefore, estimation of genotype by environment interaction effects are very important for plants instead of animals.

When the effect sizes of the genotypes are estimated simultaneously for all genes with corresponding regularization methods and the effects are summed, the sums are efficient predictions of individual genetic values. Usually, plant or animal breeders practically utilize these individual genetic values to predict the future phenotypes of plants or animals. This results in a ranking of candidate animals or plants for selection, which help breeders using prediction ahead to select the best animal lines or plant varieties to mate to save cost. There are also other methods in predicting selection candidates’ genetic values. For example, in animal or plant breeding, a mixed linear model is usually used to calculate the effect sizes of genotypes simultaneously under certain model assumption and these effect sizes are summed up for each of the selection candidates (Zhang et al., 2016a,b). Different model assumptions can be made when calculating the effect sizes, e.g., normal distribution, laplace distribution, and gamma distribution etc. (Zhang et al., 2016b; Lo and Marculescu, 2017). However, these model assumptions made are more for simplifying the mathematical treatment of the model, which does not mean a certain model assumption is always better than others.

GWAS and genomic prediction are utilized in different context in Agricultural breeding. As stated before, GWAS is more used for identify significantly associated markers to assist agricultural breeding, therefore, the markers selected from GWAS are the key information for producing SNP chips for specific species. In contrast, genomic prediction calculates the estimated breeding values in order to rank the selection candidates in practical breeding. The difference between GWAS and genomic prediction is that in GWAS, usually a single SNP is associated with the phenotype accordingly while in genomic prediction, all SNPs are simultaneously fitted in the model associating with the phenotype (Daetwyler et al., 2013; Veerkamp et al., 2016). It means that a prediction model is a comprehensive model which takes all SNPs into consideration, while a GWAS model is more focused on the association between a single SNP with different phenotypes (Zhang, 2017). In agricultural breeding, it is common to conduct various strategies of cross validations without as a supplement to formal statistical tests in order to obtain maximum accuracy with limited bias (Legarra and Reverter, 2018).

SNPs associated with phenotype that directly lead to structural changes in protein or changes and significant difference in gene expression are often called “quantitative trait loci” (QTL). However, most common SNPs are anonymous markers around the important QTLs and are in linkage disequilibrium with important QTL. Usually, there are limited numbers of significant QTLs associated with each phenotype. It is important to know how these currently detected QTLs with significant effects on phenotypes evolve during time and how the allele frequencies of QTLs are changing during time in an evolutionary perspective (Bosse et al., 2012; Purfield et al., 2012; Zhang et al., 2018). Genomic prediction is able to predict the collective effects of all the genes without even knowing the individual genes. It is of interest to know and understand how the evolutionary forces such as selection, introgression and inbreeding etc. have changed the frequency of the QTLs and how the given evolutionary constraints shape the phenotypes or the genetic architecture of the complex phenotypes during the history time. The general trend information during the history is important to infer the important genetic parameters changed through time necessarily needed for genetic improvement programs. For example, QTLs associated with a class of phenotypes are sometimes significantly enriched in genomic regions due to introgression or inbreeding (Bosse et al., 2012, 2015). It reflects that these QTLs under the demographic forces are clustered together or segregated to affect certain classes of phenotypes (Bosse et al., 2014). Moreover, some pathways or Gene Ontology (GO) terms are also enriched in a way or function together to affect the phenotypes under a certain direction during demographic processes (Bosse et al., 2015). However, in most of the cases, these QTLs or GO terms or pathways are randomly distributed across the genomes and are not significantly enriched. This suggests that these demographic processes need to have long-term effects which are strong enough to shape the genetic architectures of the phenotypes from QTLs or pathways or GO terms (Bosse et al., 2019). These types of information are very important for selection in genetic improvement programs such as putting extra weight for the specific genomic regions or loci contributing in important traits of interest especially in genomic selection to maximize genetic change per time unit.

This review article will give an overview of GWAS and genomic prediction in the context of genomics from different angles and perspectives in agriculture. It will include the importance and background of GWAS and genomic prediction in different areas such as plant and animal breeding, the generalized methods and theories, specialized methods in terms of different types of variants, the extended knowledge about GWAS and genomic prediction, and finally the applications.

## The Theories and Methods

**Figure 1:**
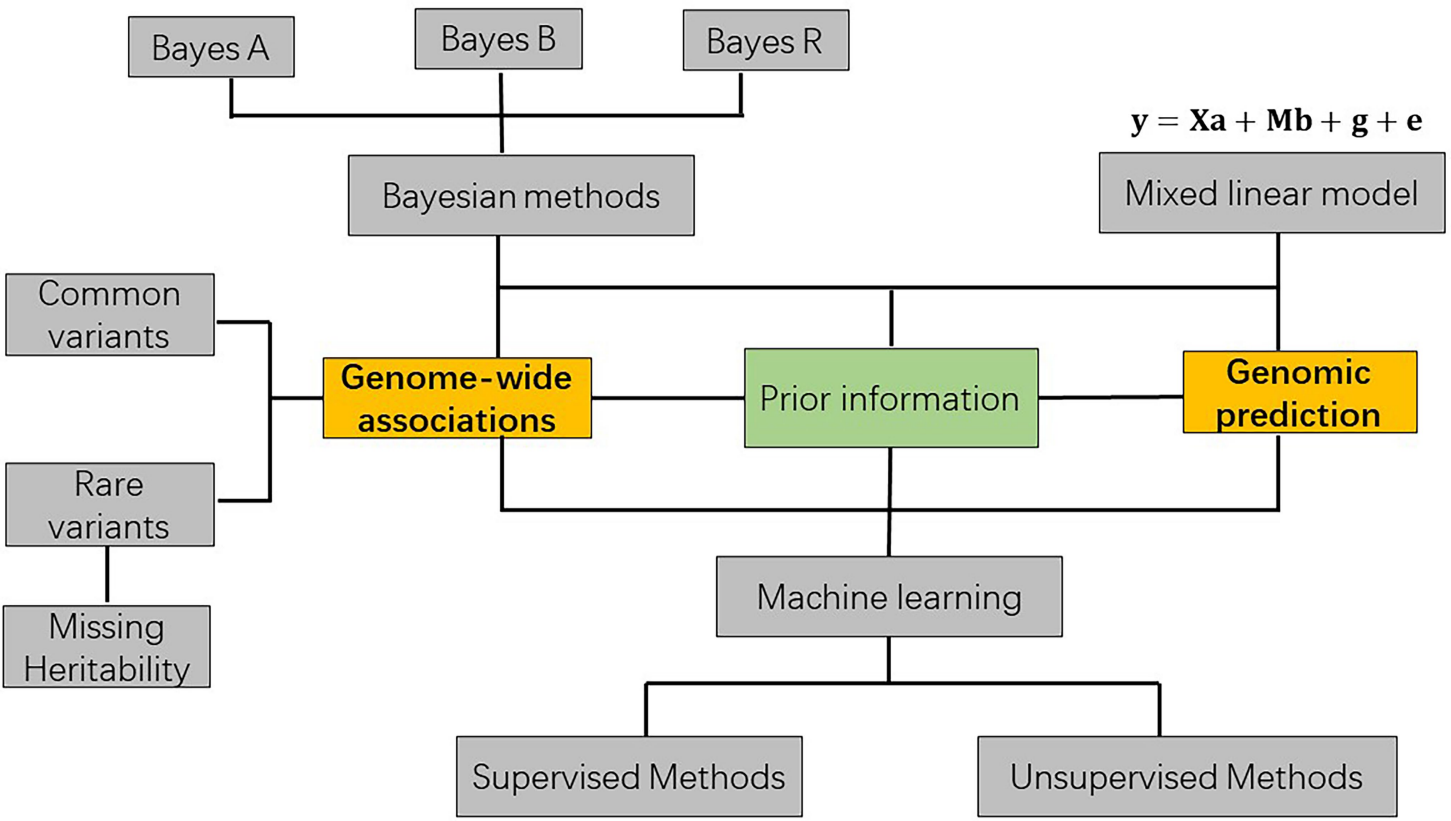
The basic statistical methods and theories for agricultural breeding.

Improving the production and performance of plants and animals with better disease resistance is the central goal for plant and animal breeding (Hammer et al., 2006; Groenen et al., 2012). Different species of animals and plants have different breeding goals depending on their use in the food chain (Daetwyler et al., 2014; Jiang et al., 2017). For example, it is of great importance to improve the production related traits and disease resistance in crops to solve the conflict between the increasing global population and lacking of major plant related food such as wheat, rice, maize etc. (FAO, 2009). GWAS and genomic prediction are the important tools in different ways to help in achieving these breeding goals in plants and animals (Daetwyler et al., 2014). GWAS can identify the potential associations between single genes and phenotypes, while genomic prediction estimates the combined effects of all genes jointly to rank selection candidates (Meuwissen and Goddard, 2010; Caballero et al., 2015; Zhang et al., 2016b). The variants associated with phenotypes identified from GWAS can be further validated whether they are causal variants or in linkage with the true causal variants (Höglund et al., 2014). It helps in understanding the genetic basis of phenotypes how different genetic variants regulating the phenotypes in different pathways (Barton and Keightley, 2002). However, GWAS and genomic prediction can be combined so that GWAS identify the strongly associated genetic variants with validating the function of QTL in a different structured population and these functional variants are expected to be emphasized in genomic prediction in the related pathways regulating the phenotypes when ranking the selection candidates in breeding (Brondum et al., 2015; Veerkamp et al., 2016). The statistical methods for GWAS and genomic prediction are more or less quiet similar and the difference between them is that the statistical method of GWAS tests the single effect of each of the genetic markers, while the statistical method of genomic prediction sum up all the markers effects in the model. The results of GWAS can provide prior information for genomic prediction (Figure 1). Generally, the statistical methods for GWAS and genomic prediction can be classified into mixed linear model (BLUP used in prediction), Bayesian methods and machine learning (Figure 1).

### Mixed Linear Models, BLUP

Here we firstly introduce the models and theories commonly used in GWAS and genomic prediction. One of the most common models for GWAS or prediction is the mixed linear model. It has been used for long time in animal and plant breeding since it was proposed in the middle of last century. The model can be written in the following general format:


y=Xa+Mb+g+e

Where **y** is a vector of observed phenotypes expected following a normal distribution, **X** is the assigning matrix for any fixed effect allocating for the specific class and **a** is covariates computed for the fixed effects, **M** is the matrix of genotypes consisting of 0, 1, and 2 corresponding to 0, 1, or 2 copies of the reference allele and **b** is the coefficients computed for the genotypic effects, **g** is the polygenic effect estimated from the pedigree or genomic relationship matrix, **e** is the random effects for random errors. We have implemented a software package for implementing the key algorithms of GWAS and genomic prediction (Zhang et al., unpublished). This model is the well-known mixed linear model that assumes a random effect for the genetic effect estimated from pedigree [also known as Best Linear Unbiased Prediction (BLUP)] or genotypes (GBLUP) with correcting on the fixed effects (Figure 1; Henderson, 1984). Many other covariance structures can be also assumed here. In mixed linear model, the additive genetic values are the sum of very many genes with very small effects. Therefore, it is by default assumed that those small effects are normally distributed due to the central limit theorem (Fisher, 1918; Hill, 2014). It is also called as infinitesimal model, i.e., assuming that there are infinite many genes with infinitesimally small effects when the number of individuals is very limited (*n* > > *p*) using in an animal model based on pedigree when no markers is available. However, the situation has changed a bit in the current age, as there are more and more genomic information collected due to the decrease of the genotyping and sequencing price. In GWAS studies, the coefficients for genetic makers are tested to determine whether it is significant or not using *t*-test or chi-square test (Sahana et al., 2010). When there are multiple number of markers tested, correction for multiple testing of value of *p* is needed. In genomic prediction, genetic effects simultaneously estimated from a mixed linear model BLUP and summed up are the estimated breeding values for each individual. However, it is very important to estimate the related parameters accurately such as genetic variance components. Commonly, the genetic variance component is derived from a joint model based on likelihood theory in which one specific parameter is estimated conditional on other parameters with maximum restricted likelihood theory in the consideration of the joint model during the iterations (Jensen et al., 1997).

### Bayesian Methods

There are also some categories of linear models such as Bayesian type of linear models (Calus et al., 2016; Zhang et al., 2020). The major assumptions of Bayesian types of models compared likelihood based models are that the genetic effects are mostly sampled from a normal distribution while sometimes other than a normal distribution (Figure 1). For example, Bayes A samples the SNP effects from a given t-distribution and this results from that a few classes of SNPs with the genetic effects from normal distributions with few degrees of freedom in the model. This Bayes B model samples the genetic effects from two component normal distributions, while Bayes R samples from a four components normal distributions (Zhang et al., 2020). The different assumptions on the distribution of genetic effects result in the different degrees of false positives and false negatives when testing the different models. These Bayesian models are based on the Bayesian theory and the models are solved by deriving the posterior probability distribution of model parameters conditional on the data available. This is often implemented using Markov Chain Monte Carlo (MCMC) methods such as the Gibbs sampler sampler to generate the random variables from the specific distributions assumed by the respective Bayesian models (Foll et al., 2008). The derived posterior probability of different variance parameters for different Bayesian models will not be presented here. Among these different Bayesian models with normal distribution assumptions, Bayes R usually performs better compared with the model only assuming one normal distribution as it assumes four components normal distributions which is more flexible (Zhang et al., 2020). This is especially important if very many markers are available and traits are affected by some genes with large effects.

### Machine Learning

Machine learning is a comprehensive set of methods for extracting and summarizing useful information using complex algorithms on big data (Figure 1; Grinberg et al., 2020). Specifically, machine learning is currently used for classification and identification of types among different agricultural varieties with a supervised or an unsupervised learning method using extracted or user specified characteristics from high-through-put data (Araus et al., 2018). In agriculture, usually only high-through-put phenotyping data is utilized due to the cost and machine learning helps in the way that the characteristics among the large amount of data can be automatically extracted to save cost. In mathematics, the large amount of data are usually complex with different correlated structure in different dimensions but these can be formatted and standardized into the matrices with different scales in the corresponding dimension. The methods of standardizing the matrices can vary which results in different accuracy of calculations compared with the other methods such as mixed linear model (Grinberg et al., 2020). Generally machine learning methods include support vector and neural network etc., which can be classified into supervised and unsupervised learning and the solutions of these models depends on the exact model assumptions in which they were applied and how the model assumptions fit the data often decides the accuracy of the model (Figure 1; Grinberg et al., 2020).

These machine learning methods have been compared with the routine methods described above such as mixed linear model and Bayesian methods in different agriculture species (Crossa et al., 2017; van Dijk et al., 2021). Machine learning can usually be used for identifying QTLs, generating a formula of the most likely genetic architecture of the studied complex traits and finally utilize these information to predict total marker values, i.e., estimated breeding values (van Dijk et al., 2021). In plant breeding, recent developments and applications were made in predicting the effects of environments, the interactive effects between genotypes and environments (Montesinos-López et al., 2018, 2019) and in both animal and plant breeding, predictions of estimated breeding values are made from secondary or in-between phenotypes utilizing the newly invented detection technologies (Cobb et al., 2013; Roitsch et al., 2019; Lopez-Cruz et al., 2020). Although mixed linear models can perform genomic prediction routinely through random effects, machine learning kind of methods still have clear advantages when the traits architecture is not normally distributed, accounting for non-additive effects such as the significant existence of dominance and epistasis (Montesinos-López et al., 2019; Abdollahi-Arpanahi et al., 2020). However, machine learning methods do not hold consistent outperformance compared with mixed linear models (Azodi et al., 2019).

## The Connections With Population Genetics

### Heritability

Heritability is a basic concept from quantitative genetics which refers to the ratio between the genetic variance and the total phenotypic variance (Zhang et al., 2017). The inference of heritability is usually based on the inferred genetic variance component and error variance component when using a restricted maximum likelihood or Bayesian theory. However, concepts of amount of genetic variance explained often depends on the method of estimation and possible misspecification of models used. It is a very important and basic concept as this reflects the genetic basis of a certain phenotype which affects the important agronomic traits in breeding. To breed the agronomic population toward a breeding goal, it is first to estimate the heritability of agronomically/economically important traits. The amount of possible genetic gain also referring to evolvability is positively correlated with heritability, genetic variance and selection intensity, while negatively correlated with generation interval. Therefore, under the same genetic variance, selection intensity and generation interval, the higher the heritability of an agronomic trait it has, more genetic gain can be possibly achieved under a breeding program. However, complex traits and diseases are often difficult to breed as they usually have a low to mediate heritability. The genetic variants usually are classified into different categories according to its allele frequencies such as common variants and rare variants. Common variants are usually defined as variants with allele frequencies of more than 0.05 while rare variants have allele frequencies of less than 0.05 (Zhang et al., 2016a). These variants with different allele frequencies have various amount of contribution on the heritability of complex traits in agriculture (Zhang et al., 2017). Generally, the number of genetic variants and their contribution to the total genetic variance reflect the genetic architecture of the different traits in agriculture.

### The Contribution of Common Variants

Genome-wide association studies have identified large amount of common variants significantly associated with different phenotypes using various models (Zhang et al., 2016b). However, these common variants associated with the phenotype are mostly anonymous markers that are linked to QTL and instead QTL very often have quite extreme frequencies. The distribution of genetic variants with effect on phenotypes typically follow a U-shaped distribution which reflects that most alleles with big effects on phenotype are un-common. Under an assumption of mixed linear model with normal distribution, the common variants collectively explain large amount of variance from the genetic variance explained in the phenotypic variance, in which a general trend is that the total genetic variance explained by QTL is proportional to the number of variants. So far, only very few common variants with large genetic effects on important traits have been found. A typical example is the common variants underlying *DGAT1* gene with big effects on milk yield (Grisart et al., 2004). However, most genetic variance in agricultural species are actually caused by numerous rare variants with small or very small effects. This makes the rare variants hard to detect individually, and therefore genomic prediction is usually utilized to estimate the summed effects from the all the genetic variants. Under an additive model the genetic variance caused by QTL can achieve the highest with the gene frequency of 0.5. This gene is common and not yet fixed in the population. In a mixed linear model, it is assumed that the effect of each genetic variant is sampled from a normal distribution with mean zero and the specific variance. It results in that most of the sampled effects will be close to zero, i.e., each QTL contributes differently in the genetic variance but the QTL effects are sampled from the same distribution (van Binsbergen et al., 2015). Therefore, when intensive selection has been performed in the agricultural populations, the allele frequency is in strong shrinkage toward zero which is more or less fixed in the population and the genetic effects become large when it is assumed that each of the variant is expected to contribute equally. Notable, this also happens in natural populations due to natural selection but at a much lower rate. In fact, many natural populations are affected by the local effective population size and therefore heavily influenced by genetic drift. Interestingly, there are still plenty of genetic variance remained in agricultural population even though under a strong directional selection through many generations. One of the challenges currently is to explain why and how the large amount of genetic variance can be maintained in a typical agricultural population. However, careful modeling is extremely important especially for the inference about natural phenomena as different model assumptions might result in different proportion and scaling of the genetic variance contributed inferred for the genetic parameters. A very careful model validation is always needed when conducting genetic analysis.

### The Role of Rare Variants

Rare variants are usually difficult to detect from genome-wide-association studies due to its low frequency and extremely small genetic effects contributed very little to genetic variance (Gibson, 2012). Several methods such as burden test and variance component test have developed which actually collapses the genetic effects of number of rare variants so that they can be detected collectively. However, in specific populations where selection has been intensively performed, the frequency of rare variants can be shaped toward a certain frequency so that they are easier to be mapped (Zhang et al., 2016a). For example, this has been the case in dairy cattle where sires with carrying recessive lethal genes have been heavily used in the international population. Otherwise, a specific alternative model or methods together using very large data sets are required to detect the effects of rare variants.

### Estimation of Heritability and the Debate About Missing Heritability

Heritability is the ratio between the total genetic variance contributed by common and rare variants and the total phenotypic variance. Estimation of the variance components usually utilize the probability theory which derives the likelihood function of the parameter with the inaccuracy around unknown genetic parameters to be estimated under the condition of other parameters and after a certain number of iterations the estimates with the best likelihood are taken as the final solution. In Bayesian estimation, we derive the posterior distribution of heritability given by data and model. Genome-wide-association studies have identified thousands of genetic variants which significantly associated with the complex traits during the recent years in agriculture (Daetwyler et al., 2014). However, these variants are mostly common variants and they collectively only explain a small amount of genetic variance contributing to heritability. There is large amount of genetic variance which has been missing in the heritability and this has been the famous mystery which has puzzled for long time for the scientists in the area of genetics (Figure 1; Gibson, 2012). To explain and solve this puzzle, scientists have come up with several arguments and try to search for the amount of missing heritability (Manolio et al., 2009). SNP chips have been used for a long time in agricultural genomics and the variants in the chips are mostly common variants often sampled with ascertainment bias. The genetic variance is mostly explained by the detected significantly associated common variants, while it is difficult to detect the rare variants using the current sequencing technology. Rare variants might plan an important role in the missing heritability problem.

## Applications

### Whole-Genome Selection for Plant and Animal Breeding

With the continued development of sequencing technology, it is possible to obtain the genotypes of different plant and animal species for the purpose of breeding. The SNP chips in different densities have been developed for many different agricultural species (Sherry et al., 2001; Brondum et al., 2015). Notably, the SNP chips only provide very few SNP compared with whole genome sequences. These commercial SNP chips can measure and test the genotypes of these agricultural species accurately and they are utilized to assist the breeding procedures in the way that significant genetic markers in linkage disequilibrium with the corresponding functional genes in phenotypes can be identified and the sum of their genetic effects can be used to rank the agricultural varieties. This owes to the long term structured mating systems used in breeding program which generates lots of short or long range linkage disequilibrium (LD) in the agricultural populations included compared with human populations that are much closer to random mating. For example, the LD of genomes of wheat varieties using as bread between adjacent loci pairs is ranging from 25.5 to 41.2 in cM with high LD *R*^2^ of 0.7 (Somers et al., 2007). The advantage of utilizing the sum of the total genetic effects is that the breeders can directly select the elite lines or varieties right after the genotypes are obtained and make decision about the mating strategies. Meanwhile, it is important that selection strategies are designed for long-term perspective and only in this way, genetic progress can be accumulated gradually toward the breeding objectives.

### Successful Examples Using Whole Genome Selection and High-Through-Put Data in Agriculture

The concept of whole genome selection, i.e., genomic selection was firstly proposed by Meuwissen et al. (2001) and it has been widely applied in different agricultural species since then. It was first applied in dairy cattle and over the last 20 years it has been a great success in dairy production (Garcia-Ruiz et al., 2016). The key of the success of genomic selection in dairy cattle is that it largely decreases the genetic interval of dairy cattle breeding process so that the genetic progress can be achieved quickly. In dairy cattle, a breeding cycle of genomic selection is typically that a reference population is firstly built up and this reference population is then both genotyped and phenotyped which are divided into training and validation set to train and validate the genomic selection model. When the genomic selection model is ready to be used, new candidate dairy cattle are tested with genotypes at an early age and their breeding values are estimated based on the genomic selection model so that they could be ranked and used for insemination for the next breeding cycle. Until now, genetic gain in different complex traits such as milk yield, protein and fat content and fertility etc. have been greatly improved, i.e., doubled or even more since genomic selection has been implemented in dairy cattle (Garcia-Ruiz et al., 2016). The similar strategy for genomic selection has also been implemented in layers compared with dairy cattle with significant reduction of generation interval and less cost of phenotyping test. Genomic selection has also been applied successfully in pig and chicken breeding, while the main advantage of genomic selection applied for pig breeding is that the accuracy for prediction of breeding values and selection of candidates is more accurate when combining phenotypic information with genomic information instead of shortening the generation interval in dairy cattle. Genomic selection has been extremely useful and powerful for improving complex traits especially for polygenic traits with many genes with small effect sizes. It has resulted in significantly improved genetic progress in animal breeding. Similarly, genomic selection can be applied in plant breeding and it has been applied in important crops breeding programs such as maize, wheat and barley breeding using combined phenotypic and genomic information. In general these methods were introduced much later in plant breeding compared to animal breeding (Zhao et al., 2012; Bassi et al., 2016; Tessema et al., 2020). In recent years, the high-through-put phenotyping (Araus et al., 2018) has been developed a lot for automatic imaging system and it has been gradually utilized more and more for agricultural phenotyping to obtain more accurate information for use in breeding programs and sometimes also to save man power.

## Implications for the Future in Agriculture

With the increasing global climate change and huge increase of the human population, there are severe problems and discrepancy between the global resources and the need of the human populations especially in places where the local population size is extremely large. People are facing these challenges and trying to solve the problems by improving the efficiency of agricultural production with keeping the balance between the environment capability and its natural resources. To meet the need of food requirement of the global population, utilizing the cutting-edge technology for breeding better breeds or varieties is the key to solve this problem.

In this review, we have summarized different cutting-edge technologies and theories including genome-wide association and genomic prediction using data collected from genomics and agronomic traits for agricultural breeding and further discussed their utilities in agricultural breeding. In the future, the output from these technologies and theories will provide the key information and knowledge for the input for the genome editing technology such CRISPR-Cas9 in crops. These cutting-edge Agricultural breeding technologies and theories are crucial for accelerating the rate of genetic progress and the key for ensuring food security for humanity. The common research topics including genome-wide-association studies and genomic prediction etc. have been discussed and we further elaborate the applications of these research topics. It reveals that the models including the algorithms behind these technologies are the core to drive these technologies. Therefore, there will be huge needs to further develop and implement these technologies and insure more and more collaboration between the different areas of research. Nowadays, the methodology have been generating large amount of data at fast speed using the current fast developing biotechnologies. This needs to be focused on data useful for improving agricultural breeding efficiency. In order to extract useful information from these large amount of data the efforts from the scientists in respective fields and multi-disciplines is needed for more efficient Agricultural breeding.

## Author Contributions

QiaZ drafted the manuscript. QinZ and JJ helped with modifying the manuscript. All authors contributed to the article and approved the submitted version.

## Funding

This work is funded by Beijing Nova Program from Beijing Academy of Science and Technology, Beijing, China (grant No. Z201100006820091).

## Conflict of Interest

The authors declare that the research was conducted in the absence of any commercial or financial relationships that could be construed as a potential conflict of interest.

## Publisher’s Note

All claims expressed in this article are solely those of the authors and do not necessarily represent those of their affiliated organizations, or those of the publisher, the editors and the reviewers. Any product that may be evaluated in this article, or claim that may be made by its manufacturer, is not guaranteed or endorsed by the publisher.
